# Case report: Traumatic carotid artery dissection after 7D High-Intensity Macro- and Micro-Focused Ultrasound treatment for skin laxity of the neck

**DOI:** 10.3389/fcvm.2022.913754

**Published:** 2022-08-18

**Authors:** Fenghe Du, Jiang Shao, Zhichao Lai, Kang Li, Chaonan Wang, Bao Liu

**Affiliations:** ^1^Department of Vascular Surgery, Peking Union Medical College Hospital, Chinese Academy of Medical Science, Beijing, China; ^2^Peking Union Medical College, Beijing, China

**Keywords:** traumatic carotid artery dissection, 7D High-Intensity Macro- and Micro-Focused Ultrasound, peripheral vascular disease, surgery, case report

## Abstract

**Background:**

Trauma is a relatively uncommon etiology of carotid artery dissection. Trauma is both penetrative and trivial, which can lead to carotid artery dissection. In the current study, we present an unusual case in which carotid artery dissection was potentially triggered by the damaging thermal effect of 7D High-Intensity Macro- and Micro-Focused Ultrasound (7D HIFU), which has been proposed as a safe and effective non-surgical modality for skin rejuvenation.

**Case summary:**

A 41-year-old woman developed headache and clinical manifestations of cerebral infarction after 7D HIFU, aimed at removing neckline. Head and neck magnetic resonance angiography (MRA) and computed tomography angiogram (CTA) revealed severe stenosis and dissection of the left internal carotid artery. Neither the patient's history nor the physical examination showed any special indicators. After resection of the left carotid artery dissection, autologous great saphenous vein interposition grafting, and simple mastoidectomy, the patient underwent head and neck MRA, which revealed recanalization of the left internal carotid artery.

**Conclusion:**

Although mild or moderate complications of 7D HIFU, such as erythema, edema, transient dysesthesia, and motor nerve paresis, have been previously reported, a few previous literature studies documented severe complications of the cosmetic procedure. However, many recent studies pointed out the possibility of 7D HIFU damaging adjacent non-target tissues due to inadequate focal depth of HIFU treatment. Our case is the first to indicate that 7D HIFU could cause carotid artery dissection. We propose that better visualization systems and more rigorous operator training are needed to reduce the risk of the potential off-target damaging effect of 7D HIFU by reporting the case in which the damaging heat effect of 7D HIFU precipitated the carotid artery dissection HIFU.

## Introduction

Carotid artery dissection, a prominent contributor to ischemic stroke in young and middle-aged patients, is caused by tearing of the arterial lining, resulting in intramural hematoma and stroke (1). Carotid artery dissection is most commonly spontaneous. The annual incidence of spontaneous carotid artery dissection is 2.5–3 per 100,000 people (1). Trauma, however, is an uncommon etiology of carotid artery dissection (2). High-Intensity Macro- and Micro-Focused Ultrasound (HIFU) has been known to be a safe and effective non-surgical treatment for skin laxity (3, 4). We present an unusual case in which the damaging heat effect of 7D HIFU was the potential trigger of carotid artery dissection in a non-elderly woman.

## Case report

**Figure 1:**
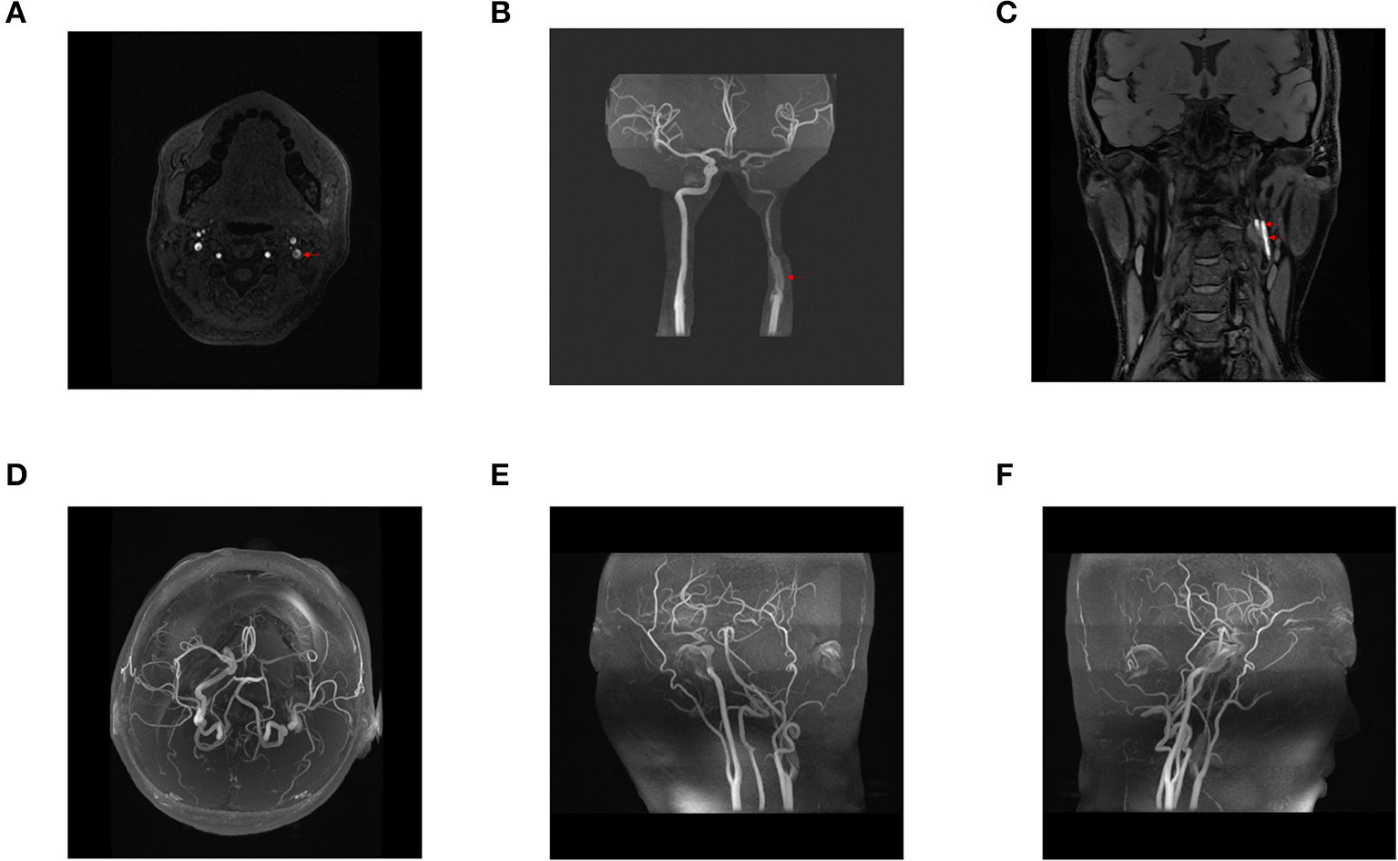
Preoperative head and neck magnetic resonance angiography (MRA), three-dimensional reconstruction of head and neck MRA, and blood flow assessment. A dissection at the origin of the left internal carotid artery was observed, and the left internal carotid artery was severely stenosed **(A–C)**. Stenosis at the origin of the left internal carotid artery was associated with an intermural hematoma **(C)**. The right internal carotid, bilateral middle, and anterior cerebral arteries showed no apparent thickening, stenosis, or signal loss **(D–F)**. The bilateral vertebral, basilar, posterior cerebral arteries and their branches were clearly displayed, with no obvious thickening and stenosis **(E,F)**. The bilateral posterior communicating arteries were found opened **(D)**. Blood flow assessment: arterial spin labeling (ASL) revealed a basic symmetry of cerebral blood flow in both cerebral hemispheres.

**Figure 2:**
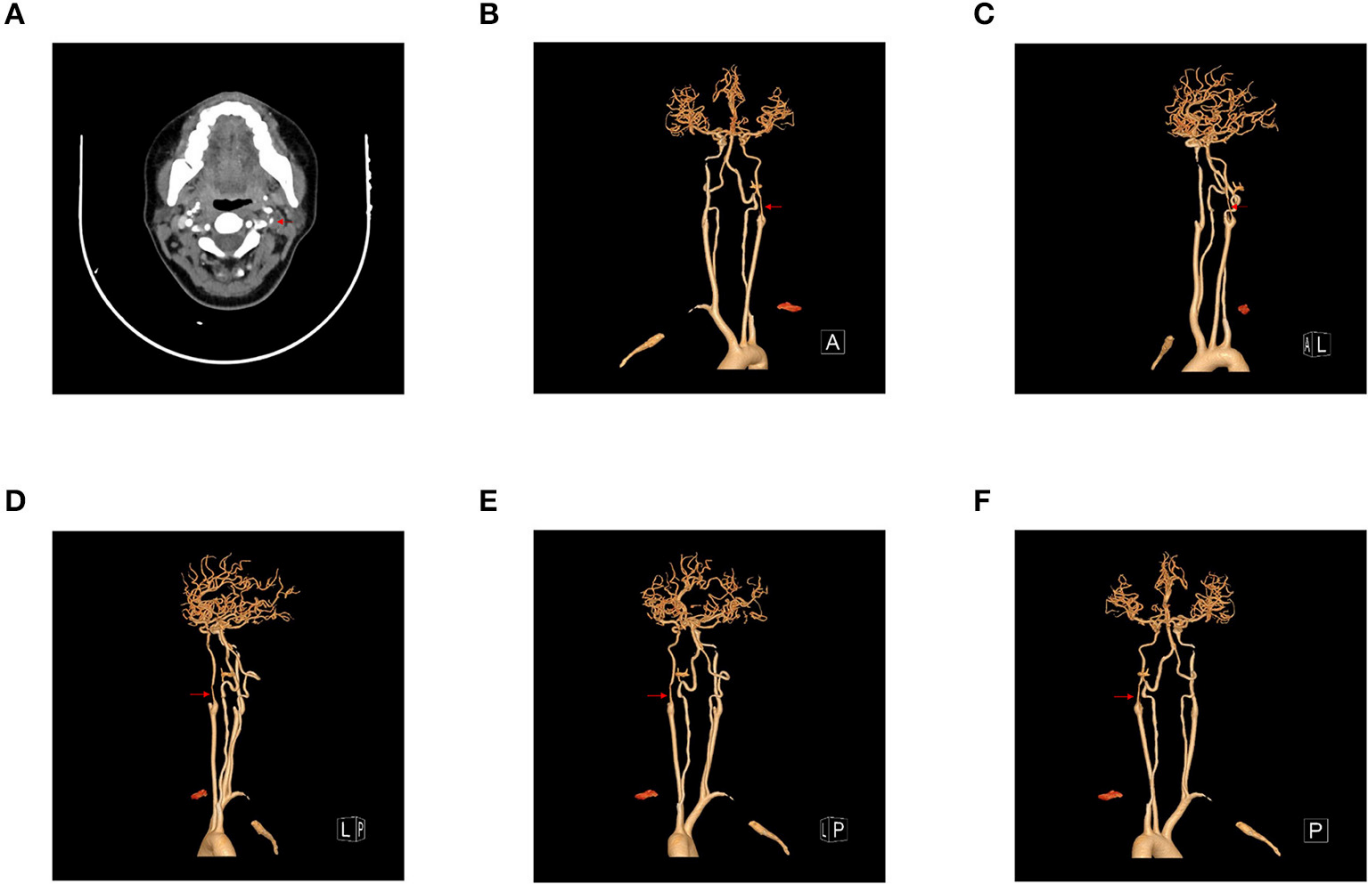
**(A–F)** Preoperative head and neck computed tomography angiogram (CTA) and three-dimensional reconstruction of head and neck CTA. Head and neck CTA showed dissection and severe stenosis at the origin of the left internal carotid artery.

A 41-year-old woman with no risk factors for cardiovascular disease and carotid artery dissection presented with speech difficulties and hemiplegic gait 2 weeks after the 7D HIFU cosmetic procedure for removing necklines. After 7D HIFU treatment, the patient had a constant, throbbing headache (NRS 2–9 points), which gradually spread from the occipital to the temporal area and was slightly alleviated by overextension of the neck. Headache was accompanied by dizziness, amaurosis, nausea, and drowsiness but was not associated with vomiting or tinnitus. Headache was not alleviated by analgesics and gradually aggravated over the past 2 weeks. MRI of the patient's head showed an ischemic stroke in the left parietal lobe, the left temporal lobe, and the left insular lobe. In addition, head and neck magnetic resonance angiography (MRA) and computed tomography angiogram (CTA) revealed dissection and severe stenosis in the left internal carotid artery (Figures 1, 2). Physical examination revealed no specific findings. We consider the damaging heat effect of the 7D HIFU to be the primary trigger of the left internal carotid artery dissection and the severe carotid artery stenosis through overall consideration of the present history, past history, physical examination, and imaging examination of the patient.

The patient was diagnosed with severe left internal carotid artery stenosis, left internal carotid artery dissection, and cerebral infarction. The patient was presented with symptomatic cerebral infarction after the 7D HIFU cosmetic procedure on the neck. Both CTA and MRA revealed carotid artery dissection accompanied by severe carotid artery stenosis. The severe stenosis of the carotid artery, most likely a complication of 7D HIFU, was the cause of cerebral infarction in this patient. The symptoms of cerebral infarction occurred within 6 months when severe carotid artery stenosis was initially detected. Therefore, carotid stenosis in this patient should be defined as symptomatic (5). According to the ESC guideline for the treatment of extracranial carotid artery disease, surgery is recommended for patients with symptomatic severe carotid artery stenosis (5). Therefore, the patient underwent resection of the left carotid artery dissection under general anesthesia.

**Figure 3:**
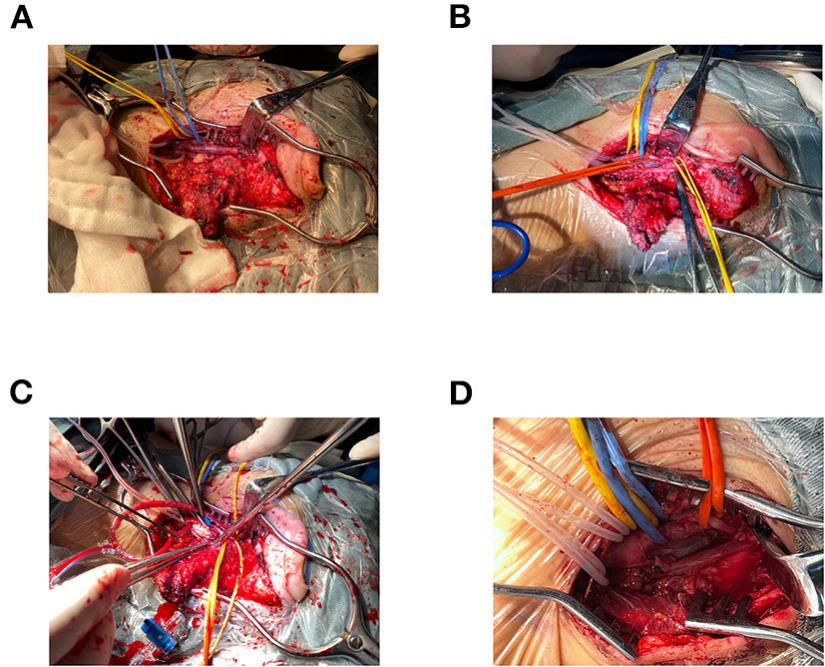
**(A–D)** Surgical procedure. The surgical procedure consisted of left carotid artery dissection resection, autologous great saphenous vein interposition grafting, and simple mastoidectomy.

**Figure 4:**
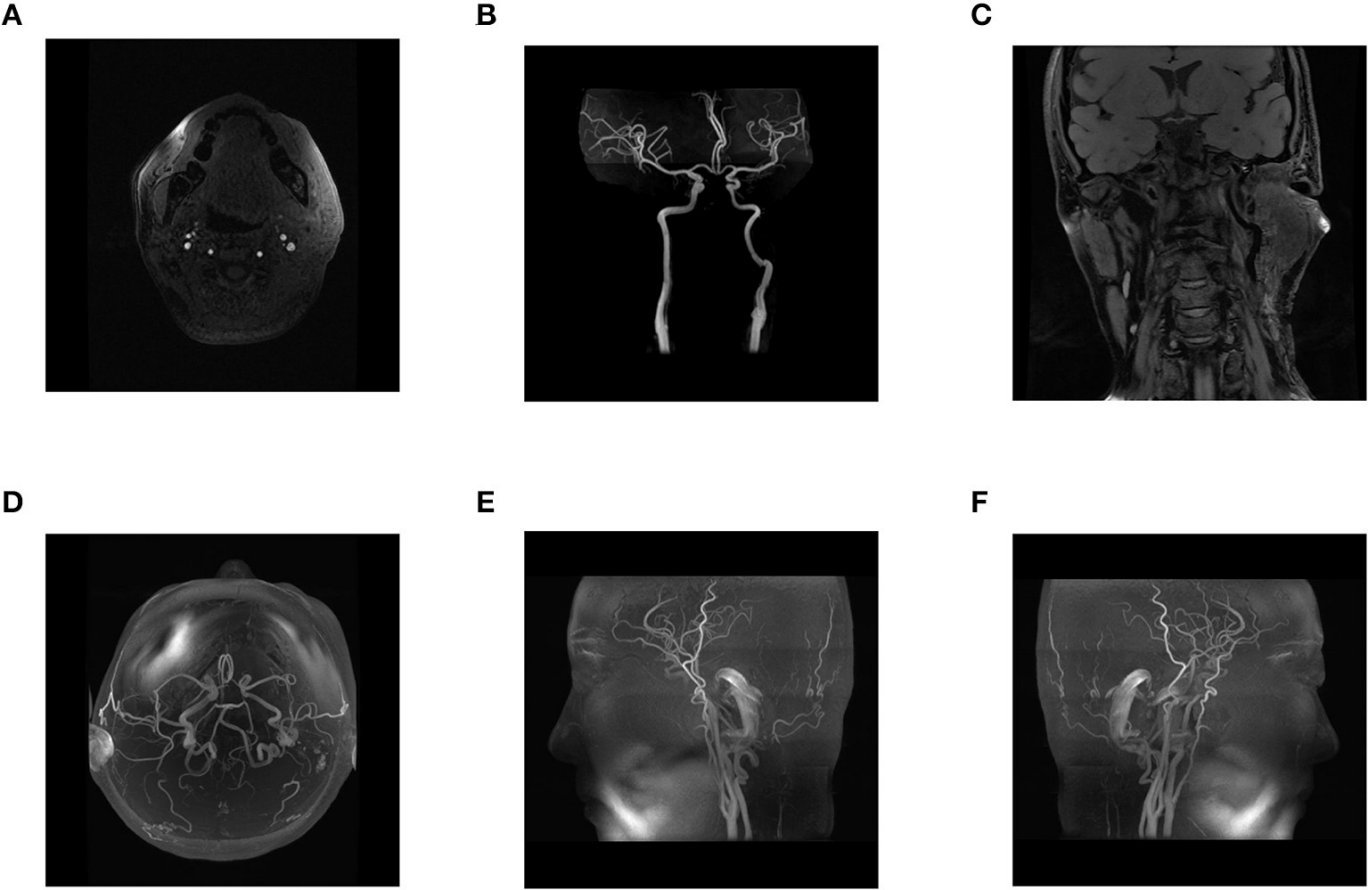
**(A–F)** Postoperative head and neck MRA, three-dimensional reconstruction of head and neck MRA, and blood flow assessment. After surgery, head and neck MRA showed recanalization of the left internal carotid artery.

After successful anesthesia, the patient was in the supine position with the head tilted to the right and the shoulders elevated. The surgical field was routinely disinfected and draped. A 5-cm oblique incision was made at the anterior edge of the left sternocleidomastoid muscle. Severe adhesion was observed in the subcutaneous fat layer. After the layer-by-layer incision, the left external and common carotid arteries were exposed, and vessel loops were placed as controls (Figure 3A). In addition to the carotid artery, the ansa cervicalis and the vagus nerve were carefully dissociated and protected (Figure 3B). Blue-purple changes of about 3 cm in length were observed on the surface of the initial segment of the left internal carotid artery, with the weak pulsation of the left internal carotid artery (Figure 3A). Since the upper pole of the pathological lesion on the internal carotid artery could not be seen, a part of the mastoid was removed. After exposing the internal carotid artery at the distal end of the styloid process, the boundary between the normal blood vessel and the diseased internal carotid artery could be identified (Figure 3B). The normal internal carotid artery was about 2 mm in diameter. While simple mastoidectomy was performed, a 5-cm longitudinal incision was made in the left groin, and the main trunk of the great saphenous vein of about 5 cm was taken. The great saphenous vein graft was fully expanded by heparin saline and placed at the internal carotid end of the bypass tube. After systemic heparinization with 5,000 units of heparin, the left common carotid artery, the external carotid artery, and the distal end of the internal carotid artery were blocked to establish bypass (Figure 3C). After performing longitudinal dissection of the lesion segment of the internal carotid artery, we observed arterial dissection, thrombus in the false lumen, complete occlusion of a part of the true lumen, and unclear intimal structure (Figure 3C). CV-6 sutures were used to perform an end-to-end anastomosis between the internal carotid artery and the great saphenous vein. After the anastomosis was successful, the diverter tube was withdrawn and exhausted, and no apparent active bleeding or oozing at the anastomotic stoma could be detected. After withdrawing the bypass tube, the left common carotid artery and internal carotid artery were blocked, and the common carotid artery-great saphenous vein anastomosis was performed using a CV-6 suture (Figure 3D). No ongoing bleeding or oozing was detected, and pulsation of the distal end of the internal carotid artery was satisfactory. The left sternocleidomastoid muscle was severed, and the surface of the mastoid was embedded. The patient returned to the ICU ward after the operation. Postoperative head and neck MRA of the patient showed recanalization of the left internal carotid artery (Figure 4).

## Discussion

The patient was a young woman who developed headache and later presented with manifestations of cerebral infarction after 7D HIFU for neckline removal. Head and neck MRA and CTA examination of the patient revealed severe stenosis and dissection of the left internal carotid artery. The patient had nothing special in terms of past history. Physical examination revealed no specific findings.

Carotid artery dissection can be spontaneous or traumatic. Spontaneous carotid artery dissection, which accounts for a significant majority of carotid dissection cases, is most commonly idiopathic, and patients often have a family history of arterial dissection and are associated with atherosclerosis, hypertension, and connective tissue disorders, including Ehlers–Danlos syndrome, Marfan syndrome, and fibromuscular dysplasia (6). However, this patient had no relevant family or past history.

Severe trauma accounts for only about 4% of carotid artery dissections (2). Traumatic dissections occur primarily in young patients and are difficult to diagnose due to the lack of symptoms in some patients or distraction from severe life-threatening injuries (7, 8). Both penetrative and blunt trauma have been reported to be the etiology of carotid artery dissection (6). Trivial trauma, including violent coughing, tooth brushing, and chiropractic manipulations, may also be a potential etiology of carotid artery dissection (6).

7D High-Intensity Macro- and Micro-Focused Ultrasound is a non-invasive treatment of skin laxity with few reported adverse effects (9). The 7D HIFU device generates high-energy-focused ultrasound that quickly penetrates the epidermis and the subcutaneous fat layer, stimulating the contraction of the membrane of the superficial musculoaponeurotic system (SMAS) layer and instantly raising the tissue temperature to 65°C. The transient increase in temperature stimulates the collagen and elastic fibers in the SMAS layer to shrink and tighten by the thermal coagulation effect, removing the wrinkles on the skin (3). Previously, mild complications of 7D HIFU, such as erythema, purpura, postinflammatory hyperpigmentation, geometrical wheals or striations, subcutaneous nodules, and edema, have been reported. Moderate complications of 7D HIFU documented in a few previous studies include transient dysesthesia and motor nerve paresis. However, severe or prolonged complications of HIFU have not yet been reported (3).

Previous animal studies demonstrated structural alterations of the whole vascular wall after HIFU treatment (10, 11). Following a session of HIFU treatment, endothelial desquamation, subendothelial edema, and leukocyte infiltration were detected in the vascular intima (10). Histological examination of the vascular media and adventitia revealed collagen shrinkage, separation, and disruption (10). The thermal coagulation effect caused by tissue absorption of HIFU mediates hyalinization and stiffening of the collagen fiber in the vascular media, making the vessel fragile and prone to rupture (12). Apart from the thermocoagulation effect, inertial cavitation, which originates from the rarefaction of HIFU, has been proposed as another critical mechanism underlying the vascular damaging effect of HIFU, as evidenced by the positive association between the severity of vascular endothelial damage and the amplitude of acoustic pressure (13). Therefore, when applied directly to the artery, HIFU could theoretically trigger arterial dissection by desquamating the arterial endothelium and stiffening the arterial wall. In addition, the structural changes in the vascular wall induced by HIFU are the foundation for subsequent stenosis or obliteration of the artery.

Prior literature has proposed the possible damaging effect of HIFU on non-target tissues, including blood vessels, as a consequence of inadequate treatment depth caused by improper transducer positioning (9). Moreover, according to a previous study, the depth of the thermal coagulation point in HIFU treatment depends not only on the power settings and exposure time but also on the types of transducers used and the thickness of the skin (14). Compared with a 4.0-mm transducer, a 6.0-mm transducer could induce a more profound and significant thermally injured area in the subcutaneous fat layer for a fixed power setting and exposure time (35 W and 90 ms, respectively). In comparison, an 8.0-mm transducer could cause unintended tissue damage under the subcutaneous skin layer (14). For the purpose of skin tightening and lifting, the appropriate focal depth of HIFU is 4.5 mm, which allows HIFU to reach the SMAS layer without penetrating beyond the SMAS layer (15–17). However, HIFU devices currently used for skin lifting and adipose reduction, including 7D HIFU devices, usually support transducers that emit various HIFU frequencies ranging from 2 to 7.5 MHz (4, 16–18). A 2-MHz HIFU transducer could penetrate to a depth of 13 mm, which increases the risk of damaging non-target tissues below the SMAS layer (18). Hence, in addition to transducer positioning, the power setting of the HIFU device, the time of exposure to HIFU, and the selection of a transducer need to be carefully considered to prevent unwanted damage to deeper layers below the SMAS layer, especially in anatomic regions, including cervical skin, where the epidermis is relatively thin and the subcutaneous layer contains less fatty tissue. In the case of carotid artery dissection, the off-target damaging heat effect of 7D HIFU on the carotid artery was the primary etiology of carotid artery dissection. Hence, we propose that image guidance and rigorous operator training are essential to ensure the safety of HIFU treatment.

## Conclusion

Previously, 7D High-Intensity Macro- and Micro-Focused Ultrasound has been considered a safe and effective non-surgical cosmetic procedure for skin lifting and wrinkle removal because no severe complications of 7D HIFU have been reported in prior literature studies. However, we report a case in which carotid artery dissection was most likely a complication of the damaging heat effect of 7D HIFU. We are the first to report that 7D HIFU has a potential risk of triggering carotid artery dissection. We propose that better visualization systems in 7D HIFU and more rigorous operator training are necessary to improve the accuracy of targeting treatment areas, thereby preventing off-target damage to adjacent tissues.

## Data availability statement

The original contributions presented in the study are included in the article/supplementary material, further inquiries can be directed to the corresponding author.

## Ethics statement

Written informed consent was obtained from the individual(s) for the publication of any potentially identifiable images or data included in this article.

## Author contributions

All authors listed have made a substantial, direct, and intellectual contribution to the work and approved it for publication.

## Funding

This work was supported by funding from the National Natural Science Foundation of China (No. 82070498), the CAMS Innovation Fund for Medical Sciences (CIFMS) (No. 2018-I2M-AI-004), the Non-Profit Central Research Institute Fund of the Chinese Academy of Medical Sciences (No. 2019XK320004), and the Fundamental Research Funds for the Central Universities (No. 3332020009).

## Conflict of interest

The authors declare that the research was conducted in the absence of any commercial or financial relationships that could be construed as a potential conflict of interest.

## Publisher's note

All claims expressed in this article are solely those of the authors and do not necessarily represent those of their affiliated organizations, or those of the publisher, the editors and the reviewers. Any product that may be evaluated in this article, or claim that may be made by its manufacturer, is not guaranteed or endorsed by the publisher.
